# Case–Parent Trio Studies in Cleft Lip and Palate

**DOI:** 10.1055/s-0040-1722097

**Published:** 2020-12-02

**Authors:** Mahamad Irfanulla Khan, Prashanth CS

**Affiliations:** 1 Department of Orthodontics & Dentofacial Orthopedics, The Oxford Dental College, Bangalore, Karnataka, India; 2 Department of Orthodontics & Dentofacial Orthopedics, DAPM RV Dental College, Bangalore, Karnataka, India

**Keywords:** cleft lip and palate, case–parent trio study, trio design, population-based studies, family-based studies

## Abstract

Cleft lip with or without cleft palate (CL/P) is one of the most common congenital malformations in humans involving various genetic and environmental risk factors. The prevalence of CL/P varies according to geographical location, ethnicity, race, gender, and socioeconomic status, affecting approximately 1 in 800 live births worldwide. Genetic studies aim to understand the mechanisms contributory to a phenotype by measuring the association between genetic variants and also between genetic variants and phenotype population. Genome-wide association studies are standard tools used to discover genetic loci related to a trait of interest. Genetic association studies are generally divided into two main design types: population-based studies and family-based studies. The epidemiological population-based studies comprise unrelated individuals that directly compare the frequency of genetic variants between (usually independent) cases and controls. The alternative to population-based studies (case–control designs) includes various family-based study designs that comprise related individuals. An example of such a study is a case–parent trio design study, which is commonly employed in genetics to identify the variants underlying complex human disease where transmission of alleles from parents to offspring is studied. This article describes the fundamentals of case–parent trio study, trio design and its significances, statistical methods, and limitations of the trio studies.

## Introduction

Cleft lip with or without cleft palate (CL/P) is one of the most common congenital birth defects with a complex etiology, 1 involving various genetic and environmental risk factors. 2 3 4 The prevalence of cleft lip and palate ranges from 1 in 700 to 1,000 newborns worldwide. Its prevalence is lowest in Africans (1:2,500), average in Caucasians (1:1000), and the highest in East Asians (1:500). 5 Advances in genetics and molecular biology have explored the genetic basis of development of these craniofacial defects. 6 7 8 Genetic studies aim to understand the mechanisms contributory to a phenotype by measuring the association between genetic variants and also between genetic variants and phenotype population. Genome-wide association studies (GWASs) are standard tools used to discover genetic loci related to a trait of interest. 9 The underlying concept for GWASs is to perform a test of association for every single-nucleotide polymorphisms (SNPs) across the genome and then examine the regions showing the most statistical significance. 10

Genetic association studies are generally divided into two main design types: population-based studies and family-based studies. 11 The population-based studies comprise unrelated individuals that directly compare the frequency of genetic variants between cases and controls are widely used for association studies. 12 The goal of these studies is to detect potential genetic loci with a different frequency between cases and controls, which can correspond to conferring disease risk. Case–control designs are increasingly being employed for GWASs 13 because of the convenience in the recruitment of samples and also the reducing cost of genotyping large numbers of individuals. However, the ensuing analyses are vulnerable to false correlation arising from population stratification. 14

The alternative to population-based studies (case–control designs) includes various family-based study designs that comprise related individuals. 15 16 17 18 An example of such a study is case–parent trio design study, where transmission of alleles from parents to offspring is studied. 19 20 21 22 It is also known as the “case–parental control” or the “triad” design or the “trio design” study. 23 24

## Trio Design

**Fig. 1:**
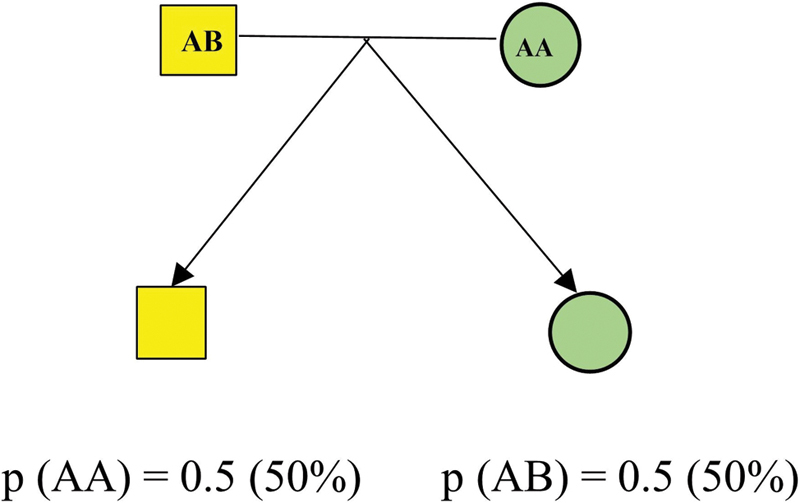
Trio design.

The model of Mendelian inheritance offers a straightforward rationalization of the genetic design of a trait. It prescribes that one gene locus produces the trait in either recessive or dominant pattern in families. However, many traits do not follow such a straightforward model of genetic architecture. Based on Mendelian inheritance principles, every parental allele has a 50% probability of being transmitted. 25 26 The basic trio design is shown in Fig. 1 .

Under the null hypothesis of no association between the disease and the marker, every parent transmits one of their two alleles to each offspring at random with a probability of 50/50 and independently of the other parent and any other oﬀspring. For the example in Fig. 1 , the mother can only transmit the A allele. However, the father can transmit either A or B with a probability of 50/50. This holds whenever there is no selection of the oﬀspring related to the marker in question. Thus, when the parent's genotypes are known, one can easily calculate the distribution of the oﬀspring genotypes under the null hypothesis. 27

## Case–Parent Trio Design

Case–parent trio studies are commonly employed in genetics to identify the variants underlying complex human disease. It involves the diseased/affected child and their parents (father and mother). In these studies, the affected children are selected from a population and then the affected children and their parents are genotyped. 28 Therefore, it does not require control sample data necessarily because the nontransmitted parental alleles or genotypes are used as “controls” for the transmitted alleles or genotypes. 29

Case–parent trio designs study the effects of a gene marker and gene–environment (GxE) interaction. 30 They are helpful to study the transmission of genetic variants between parents and offspring and how genetic variants differ between the affected individual(s) and the unaﬀected individuals within a family. 31 32 Trio design studies are popular and an alternate to population-based case–control studies where unrelated individuals are being used for the detection of variants underlying common complex disease risk. Case–parent trio study designs also guard against population stratification and therefore the resulting type I error inflation, which is commonly observed in population-based studies. This technique is most robust to population admixture, and the targeted sequencing of trios is powerful to identify genetic variants that alter the function of the gene. 33 34

The family-based association studies were initially proposed by Falk and Rubinstein to avoid false-positive association test results. 35 They collected the parental genotypes of every case used the nontransmitted parental alleles as a control sample because the cases and controls are matched in genetic ancestry and are therefore robust to population stratification. 36 Later these tests were developed by other authors Ott (1989), and Terwilliger and Ott, (1992). Furthermore, Spielman et al proposed “transmission disequilibrium test” (TDT) to identify preferential transmission of alleles from parent to the affected child. 37 38 39 40

In most of the genetic association studies, investigators compare an allele or genotype frequencies in unrelated case and control subjects or examine preferential allele transmissions from parents to affected offspring. It is important to contemplate parent of origin effects 41 while studying congenital birth defects such as CL/P because maternal genotype controls the in utero environment of the developing fetus and separates maternal genotypic effects from imprinting effects. 42 43 The functional activity of some genes or chromosomal regions depends on whether they are transmitted maternally or paternally, and this epigenetic phenomenon is termed as *genomic imprinting* . 44 45 Another commonly used method in case–parent trio study is to use the non-transmitted genotypes of parents to affected offspring as control (also known as pseudo controls or complements). 46

GxE interactions have significant scientific and public health implications. 47 48 49 In the absence of GxE interactions, 50 using the affected individual and their parents has been proved to be the most robust method to assess disease associations with candidate genes, also as an assessment of both linkage and allelic association with genetic markers. 51 The aim of collecting the genetic data from family members aids in (1) enriching the genetic analysis, (2) increasing the power of study, (3) obtaining correct mutations, (4) addressing the priority regarding population structure bias, and methods of mixing genetic information from unrelated cases and controls. 21 52

This design has the most significant advantage of being robust to population structure bias as the estimation of gene transmission would be within the families, conditioning on parental genotypes, and it is an unbiased method as all the subjects are from the same family. 53 Additionally, family data are always advantageous because certain models will test for maternally mediated and parent-of-origin effects that are significantly relevant when studying congenital birth defects in humans. 54

### Case–Parent Trio Design Significances

Several genetic studies focused on numerous complex genetic and epigenetic effects associated with a nonsyndromic CL/P, suggesting that the mother transfers half of her genome to the offspring and provides the environment for the fetus. Variation within the mother's genome could affect the intrauterine environment to the development of the fetus. Parent-of-origin effects, where the effect of inherited DNA depends on whether it is transmitted from the mother or the father, may be difficult to study with a population-based case–control design. More complex genetic effects such as maternal genotype effects, maternal–fetal interactions, and parent-of-origin effects can be best studied using the case–parent trio design, where affected cases and their parents are genotyped. 55 56

#### Advantages of Case–Parent Trio Design Studies

The advantages of case–parent trio design studies are as follows 15 17 20 21 54 57 :

- Robustness in sample collection and false conclusions because of population stratification and ethnic heterogeneousness.
- Testing directly for maternal versus paternal effects.
- It permits the effects of fetal genotype versus parental origin in a very robust manner.
- Correct mutations are often obtained.
- It is unbiased as they share the same genetic ancestry.
- Simplex families (only the affected member) will be used effectively to check for linkage in the presence of disequilibrium.
- Family information will be helpful to check maternal genotype and parent-of-origin effects.
- It minimizes problems with confounding that plague traditional case–control designs as a result of the observed case is always compared with “controls” obtained from the same family.
- It is useful to check for GxE interaction under the conditional logistic or logistic regression framework.

## The Statistical Methods for Case–Parent Trio Studies

Case–parent trio design studies employ a distinct statistical procedure to examine the linkage and association of a gene with the trait. When studying families, the transmission of alleles from the parent to the offspring is analyzed. In this sense, parents are used as genetic controls for their children. 38 58 59 60 61 62 There are three main methods in which trios are analyzed, namely; the Transmission Disequilibrium Test (TDT), Conditional on parental genotype (CPG) approach, and combined likelihood method: 63 64 65 66 67

- *Transmission Disequilibrium Test (TDT)* : it compares the offspring to its antiself, and the unobserved instance of an offspring with each nontransmitted allele.
- *Conditional on parental genotype (CPG)* : it compares the proband to unobserved pseudosiblings with all possible transmission patterns. Again, these pseudosiblings are not determined; however, the results of conditioning on the parental genotype are used to make a matched analysis.
- *Combined likelihood approach* : this method collectively models the likelihood of parental and case genotypes.

The most common statistical procedure used in case–parent trio design study is the TDT introduced by Spielman et al. It is a robust test for the linkage and disequilibrium in a variety of complex diseases that are associated with genetic etiology. It is more sensitive than haplotype sharing tests and requires only simplex families. 68

The CPG approach developed by Schaid and Sommer models the probability of an affected child's genotype CPG as a function of the genotype relative risks (GRRs) of the child. 69 70 This CPG likelihood approach permits versatile modelling of the GRRs, which may be quantifiable using standard maximum-likelihood procedures. GRR is also helpful in estimating the magnitude of the gene–disease association in case–parent trio studies. 71 72

There are various commercially available software suites for case–parent trio study designs such as Golden Helix ( http://www.goldenhelix.com ) and freely accessible software system PLINK. 73 74 PLINK is arguably the most commonly used software and a very powerful environment to carry out genetic association studies. It includes an integrated module to carry out the allelic TDT introduced by Spielman et al to assess the marginal SNP effects on the phenotype in case–parent trio studies.

Unlike the case–control approach, family-based methods are not subject to inflation of results because of population substructure, as they examine the transmission between parents and offspring and remove any potential impact caused by population allele frequency variations. However, family-based methods have less statistical power than the population-based case–control designs for a similar number of individual studied. 75

### Disadvantages of Case–Parent Trio Design Studies

The disadvantages of case–parent trio design studies are as follows: 20 48 52 76 77 78 79

- Not all case–parent trios are informative (only heterozygous parents give information), some are intrinsically uninformative about allele transmission.
- They provide a robust assessment of gene effects; however, they do not impart complete information on GxE interaction.
- Not all of the parental genotypes (one or both) are available for the study because of death, separated parents (divorced), or false paternity.
- Some families/parents may refuse to participate in clinical research.
- The cost of genotyping is expensive for the case–parent trios (three persons) than for the case–controls (two persons) without a corresponding linear increase in statistical power.
- Difficulty in recruiting large samples and time-consuming.
- Discarding those families with missing one or both parental genotypes will cause statistical power loss.
- If there is no deviation from multilocus Hardy–Weinberg equilibrium, the case–parent trio design will have no power to discover even tight linkage.

## Conclusion

Case–parent trio studies are commonly used in genetics to identify the variants underlying complex human disease. They are useful to study the transmission of genetic variants between parents and oﬀspring and how genetic variants diﬀer between the aﬀected individual(s) and the unaﬀected individuals within a family. This design has the most significant advantage of being robust to population structure bias and can test for more complex genetic effects such as maternal genotype effects, maternal–fetal interactions, and parent-of-origin effects that are significantly relevant when studying congenital birth defects.

In the absence of GxE interactions, using cases and their parents has proved to be a powerful test to assess disease associations with candidate genes. Case–parent trio study designs are adequate to estimate the genetic effects, and therefore the aim of collecting genetic information from family members enrich the genetic analysis, increase the power of study, help in obtaining correct mutations, and address the priority regarding population structure bias and methods of combining genetic information from unrelated cases and controls.
